# Human breast cancer-derived soluble factors facilitate CCL19-induced chemotaxis of human dendritic cells

**DOI:** 10.1038/srep30207

**Published:** 2016-07-25

**Authors:** Hyundoo Hwang, Changsik Shin, Juhee Park, Enoch Kang, Bongseo Choi, Jae-A Han, Yoonkyung Do, Seongho Ryu, Yoon-Kyoung Cho

**Affiliations:** 1 School of Engineering and Sciences, Tecnologico de Monterrey, Campus Monterrey, Ave., Eugenio Garza Sada 2501 Sur, Monterrey, NL 64849, Mexico; 2 Center for Soft and Living Matter, Institute for Basic Science (IBS), UNIST-gil 50, Ulsan 44919, Republic of Korea; 3 Department of Bio and Brain Engineering, KAIST, 291 Daehak-ro, Yuseong-gu, Daejeon 34141, Republic of Korea; 4 School of Life Sciences, Ulsan National Institute of Science and Technology (UNIST), UNIST-gil 50, Ulsan 44919, Republic of Korea; 5 Soonchunhyang Institute of Medi-bio Science (SIMS), Soonchunhyang University, Chonan-Si Chungcheongnam-do 31538, Republic of Korea

## Abstract

Breast cancer remains as a challenging disease with high mortality in women. Increasing evidence points the importance of understanding a crosstalk between breast cancers and immune cells, but little is known about the effect of breast cancer-derived factors on the migratory properties of dendritic cells (DCs) and their consequent capability in inducing T cell immune responses. Utilizing a unique 3D microfluidic device, we here showed that breast cancers (MCF-7, MDA-MB-231, MDA-MB-436 and SK-BR-3)-derived soluble factors increase the migration of DCs toward CCL19. The enhanced migration of DCs was mainly mediated via the highly activated JNK/c-Jun signaling pathway, increasing their directional persistence, while the velocity of DCs was not influenced, particularly when they were co-cultured with triple negative breast cancer cells (TNBCs or MDA-MB-231 and MDA-MB-436). The DCs up-regulated inflammatory cytokines IL-1β and IL-6 and induced T cells more proliferative and resistant against activation-induced cell death (AICD), which secret high levels of inflammatory cytokines IL-1β, IL-6 and IFN-γ. This study demonstrated new possible evasion strategy of TNBCs utilizing their soluble factors that exploit the directionality of DCs toward chemokine responses, leading to the building of inflammatory milieu which may support their own growth.

The tumor microenvironment is the site where tumors develop through complicated interactions between tumor cells and various cells within or surrounding tumor nests, such as stromal cells, innate immune cells, and lymphocytes^1^. Complex multi-factors derived from the tumor microenvironment and various immune responses in the tumor microenvironment can often influence clinical outcomes, resulting either in tumor rejection or tumor promotion^2^,^3^. Since dendritic cells (DCs) play a key role in the initiation and regulation of antigen-specific immune responses^4^,^5^, the roles of DCs in the tumor microenvironment and their immune consequences have been actively investigated^6^. Tissue-resident immature DCs continuously take up and process antigens, and upon encountering danger signals, these antigen-bearing DCs undergo a maturation process expressing CCR7 and migrate responding to their ligand CCL19 or CCL21 into the T cell enriched-area of secondary lymphoid organs^7^. Here, the mature DCs present the processed antigens to T cells, leading to T cell priming and differentiation^8^. Thus, DCs play a major role as a sentinel in cancer immunosurveillance and are capable of initiating anti-tumor immune responses. In fact, the infiltration of DCs into primary tumor lesions has been associated with prolonged survival and better clinical outcomes in many different types of cancers^9^. In contrast, altered maturation and dysfunctional DCs have been reported to occur in cancer patients and diverse strategies of tumors to evade, suppress, or tolerate the anti-tumor activity of DCs have been proposed^10^,^11^.

A deficiency in mature DC (mDC) infiltration into primary breast tumor sites and dysfunctional DCs isolated from breast cancer patients have also been reported^12^, which may explain the poor immunogenicity of breast cancers. Immature CD1a^+^Langerin^+^ DCs have been found within these tumors, whereas mature CD83^+^Lamp^+^ DCs have been located in peritumoral areas^13^. Moreover, deficiency of the active form of DCs has been observed in the breast cancer microenvironment^14^. When DCs were isolated either from peripheral blood or lymph nodes of breast cancer patients, impaired maturation and reduced T cell stimulatory activity of DCs were observed^15^,^16^ and these cells were more susceptible to spontaneous apoptosis in comparison to those from healthy volunteers^17^. Nonetheless, unlike other tumor types, no positive correlation between infiltration of DCs in the breast cancer microenvironment and survival of cancer patients or better outcomes have been reported^18^,^19^,^20^. These discrepant findings prompted us to investigate the unknown effects of breast cancer-derived factors on the migratory features of DCs followed by their ability in inducing T cell immune responses. In addition, studies reported that the main cause of death was not due to primary breast cancers but due to their metastases at certain sites^21^,^22^. However, it has not been clearly explained whether there are altered functions or migratory properties of DCs particularly by breast cancers-derived factors.

In this study, we utilized single cell-based analysis in a 3D microfluidic device and observed that soluble factors secreted from breast cancer cell lines increased CCL19-induced directional persistence of human DCs. We found that the triple negative breast cancer (TNBC) cells such as MDA-MB-231 and MDA-MB-436 facilitate the CCL19-induced directional movement of human DCs via highly up-regulated the JNK/c-Jun signaling pathway. We also demonstrated that TNBC-affected DCs secrete pro-inflammatory cytokines and T cells that have been induced by these DCs are highly proliferative and are resistant to activation-induced cell death (AICD) while secreting profound pro-inflammatory cytokines, building host inflammatory milieu that may support tumor growth.

## Results

### 3D microfluidic device was utilized to examine the chemotaxis and chemokinesis of DCs

**Figure 1:**
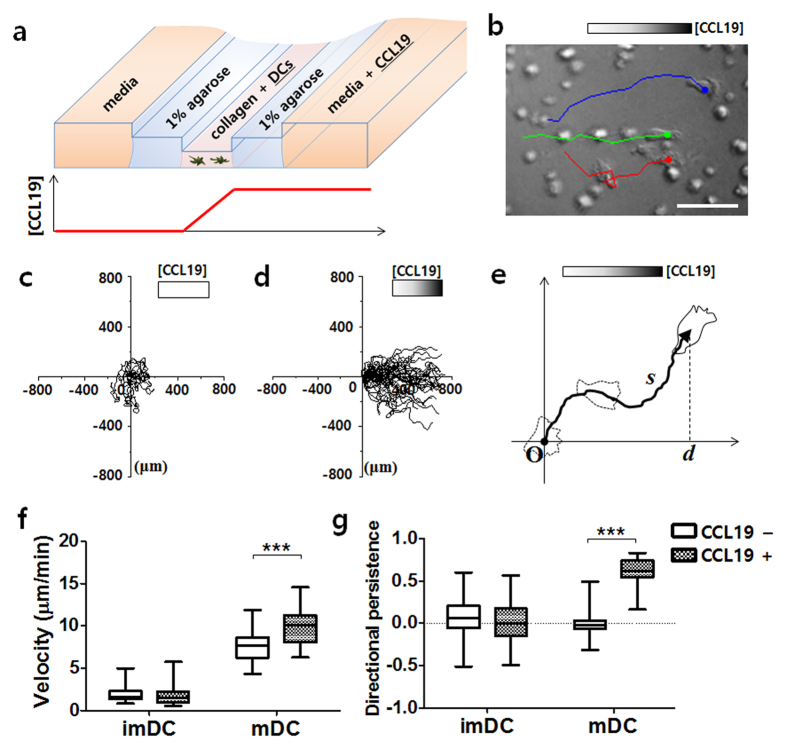
Migration of DCs under the CCL19 concentration gradient in the 3D microfluidic device. (**a**) Schematic view of a multilevel microfluidic device for a cell migration assay. A linear concentration gradient of CCL19 is formed across the cell-laden collagen gel. (**b**) Bright line images of mature DCs (mDCs) migrating toward CCL19. Trajectories of three cells over a period of 20 min are overlapped. Scale bar = 50 μm. (**c**,**d**) Trajectories of mDCs in the absence (**c**) or presence (**d**) of CCL19. *n* = 40. The starting position of each cell is superimposed. The cells were imaged every 2 min and tracked for 2 h. (**e**) The cellular trajectory (solid arrowed line) was analyzed to calculate the velocity (*s*/*t*) and directional persistence (*d*/*s*) of the migrating cells, where *s* is the total migration distance, *d* is the displacement along the gradient axis, and *t* is the time. (**f**,**g**) Box-and-whisker plots of the velocity (**f**) and directional persistence (**g**) of immature DCs (imDCs) or mDCs. Data were calculated from the cellular trajectories. *n* ≥ 40. ***P *<* 0.001.

To investigate whether the chemotaxis or chemokinesis of DCs are influenced by human breast cancer-derived soluble factors, a 3D microfluidic device was applied to construct an *in vitro* microenvironment under a stable chemical concentration gradient (Fig. 1a). Microchannels comprising three different levels were fabricated by molding poly(dimethylsiloxane) (PDMS) with a multilayer-stack of plotter-cut adhesive tapes (Supplementary Fig. S1)^23^. This multilevel channel design provides substantial advantages for on-chip cell migration assays—such as cleanroom-free device fabrication and pipette-based sample delivery. In addition, tracking the movements of individual cells in the microfluidic device allowed us to analyze their migration quantitatively, thus providing more detailed information about cellular behavior, which cannot be obtained by using traditional transwell migration assays^24^.

Here, 1 μL collagen gel, containing DCs, was loaded into the shallowest main channel, while two side-channels adjacent to the main channel were filled with 1% agarose gel. The human monocyte-derived DCs were prepared and matured with 25 μg/ml poly (I:C), a DC maturation stimulus, before loading into the chip. A linear concentration gradient of CCL19 was formed across the collagen gel by loading a 3 μg/ml CCL19 solution into the media chamber (Fig. 1a). The gradient of CCL19 within the collagen gel was expected to be around 1.8 μg/ml per mm, as determined by numerical simulation (Supplementary Fig. S2). The positions of individual DCs were recorded every 2 min and were tracked for 2 h (Fig. 1b). The mDCs tended to migrate toward the high CCL19 concentration of the gradient, while they showed random movements in the absence of the gradient (Fig. 1c,d). From the cell trajectory data, the velocity and directional persistence of each cell could be calculated quantitatively as *s*/*t* and *d*/*s*, respectively, where *s* is the total distance of the traveled cell, *t* is the total time the cell was tracked, and *d* is the displacement along the gradient axis (Fig. 1e). ImDCs were devoid of directionality, regardless of the presence of the CCL19 gradient. Both the velocity and directional persistence of mDCs were significantly increased in the presence of the CCL19 gradient (Fig. 1f,g). Based on these results, we confirmed that CCL19 induces both chemokinesis and chemotaxis of human mDCs, which is consistent with the previous studies^25^,^26^. Our results also indicated that the multilevel microfluidic device offers reliable quantitative data collection, and thus is applicable for the study of the effect of breast cancer-derived soluble factors on CCL19-induced DC chemotaxis.

### Breast cancer-derived soluble factors facilitate the directional movement of DCs toward CCL19

**Figure 2:**
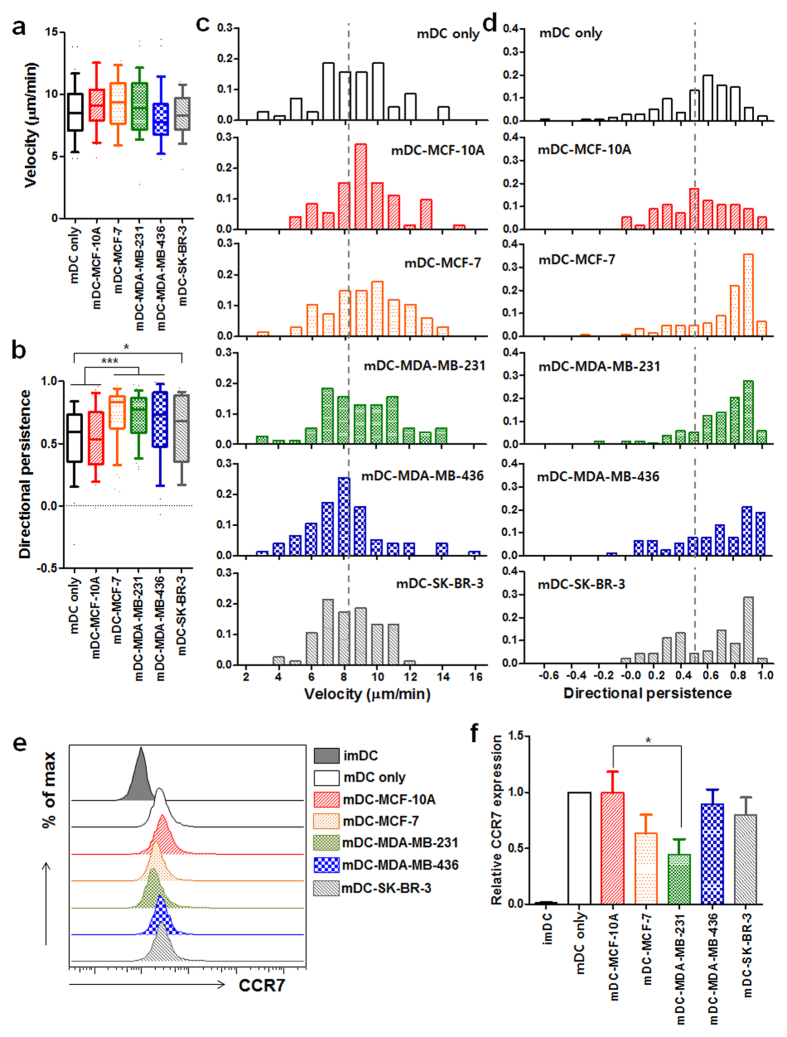
Enhanced directional movement of breast cancer-affected mDCs toward CCL19. (**a**,**b**) Box-and-whisker plots of the velocity (**a**) and directional persistence (**b**) of mature DCs (mDC only) or mDCs co-incubated with MCF-10A (mDC-MCF-10A), MCF-7 (mDC-MCF-7), MDA-MB-231 (mDC-MDA-MB-231), MDA-MB-436 (mDC-MDA-MB-436) or SK-BR-3 (mDC-SK-BR-3) in response to CCL19. (**c,d**) Histograms of the velocity (**c**) and directional persistence (**d**) of mDC only, mDC-MCF-10A, mDC-MCF-7, mDC-MDA-MB-231, mDC-MDA-MB-436 or mDC-SK-BR-3. The mean velocity and directional persistence of mDC only are indicated by a dotted line. *n* ≥ 70. (**e**) Flow cytometric analysis of CCR7 expression on the indicated groups. (**f**) Data represent mean ± s.e.m. of at least three independent experiments described in (**e**). Data was normalized by mDC only (value = 1). *P < 0.05 and ***P < 0.001.

To investigate the effects of human breast cancer-derived factors on DCs, we utilized four different types of breast cancer cell lines: epithelial MCF-10A; luminal MCF-7; two different metastatic TNBC cell lines, MDA-MB-231 and MDA-MB-436; and HER2-positive SK-BR-3. Human peripheral blood monocyte-derived immature DCs (imDCs) were co-cultured each of various breast cancer cell lines in the presence of poly (I:C). Each human breast cancer cell line was seeded on the culture plate 12 h before the co-culture under the membrane insert with 0.4 μm pores on which imDCs were loaded, designed for only soluble factors to freely move. In the presence of poly (I:C), all DCs co-cultured with various breast cancer cells undergone normal maturation processes based on highly expressed co-stimulatory molecules, CD80, CD83 and CD86, and HLA-DR when compared with those expressed on mature DCs (mDCs) co-cultured with MCF-10A (mDC-MCF-10A) (Supplementary Fig. S3), although HLA-DR expression on mDC-MCF-10A was higher. We from here designated mDCs indirectly co-incubated with each of various cell lines as mDC-MCF-10A, mDC-MCF-7, mDC-MDA-MB-231, mDC-MDA-MB-436, or mDC-SK-BR-3. 2 days after the co-culture, we purified mDCs, and examined their chemotaxis and chemokinesis toward CCL19 in our 3D microfluidic device. Interestingly, we observed that the overall chemotaxis of mDCs co-cultured with breast cancer cells was significantly increased while no such enhanced chemotaxis was observed from mDC-MCF-10A compared with that of mDC only. Their enhanced chemotaxes were mainly mediated via significantly enhanced directional persistence (Fig. 2b,d), while the migration velocity was comparable in all groups (Fig. 2a,c). In addition, the increase in directional persistence of mDCs interacted with luminal (mDC-MCF-7) or TNBC cell lines (mDC-MDA-MB-231 and mDC-MDA-MB-436) was more significant than that of mDCs co-incubated with HER2-overexpressing SK-BR-3 (mDC-SK-BR-3) (Fig. 2b,d). Since the culture condition of MCF-10A cell requires additional substances such as insulin, epidermal growth factor (EGF) and hydrocortisone, and insulin and hydrocortisone are known to alter DC maturation and function^27^,^28^, we utilized an conditioned medium for maintaining all cell lines and DCs, and found no significant effects of the conditioned medium on the migratory properties of mDCs (Supplementary Fig. S4).

### Enhanced chemotaxes of mDC-MDA-MB-231 and mDC-MDA-MB-436 were mediated by a highly up-regulated JNK/p-c-Jun signaling pathway

**Figure 3:**
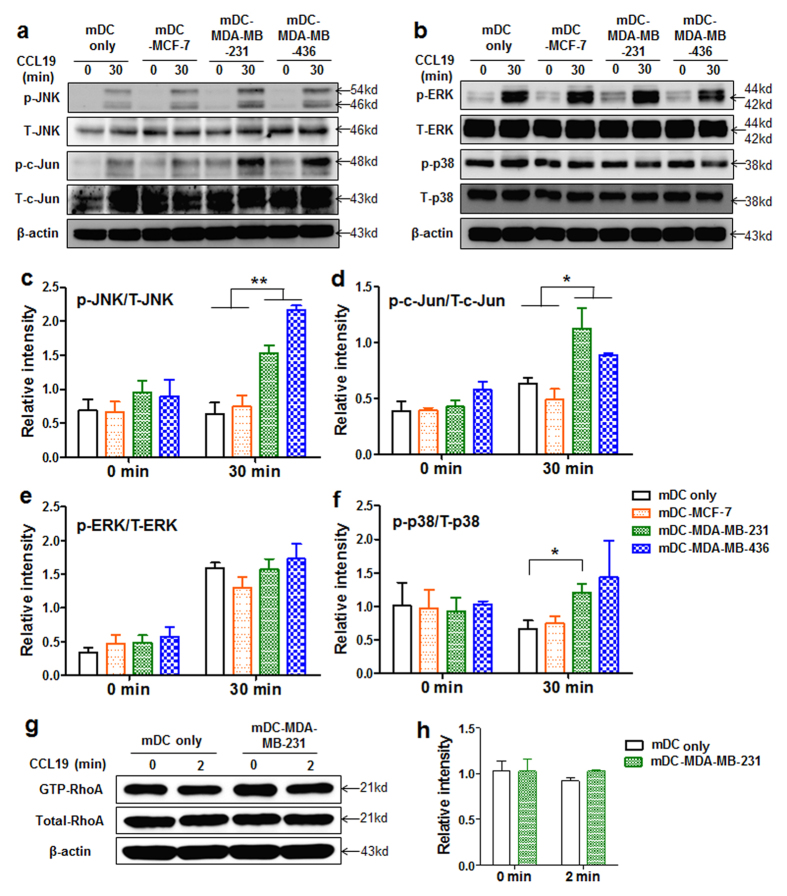
CCL19 induced JNK/c-Jun signaling pathway is highly activated in mDC-MDA-MB-231 and mDC-MDA-MB-436. (**a**,**b**) Expression of MAP kinase molecules, JNK, c-Jun, ERK or p-38, in mDC only, mDC-MCF-7, mDC-MDA-MB-231 or mDC-MDA-MB-436 at 0 or 30 min after CCL19 treatment. (**c**,**f**) Data represent mean ± s.e.m. of the ratio of p-JNK/T-JNK (**c**), p-c-Jun/T-c-Jun (**d**), p-ERK/T-ERK (**e**) or p-p38/T-p38 (**f**) from at least three independent experiments described in (**a,b**). (**g**) Activity of GTP-RhoA in mDC only or mDC-MDA-MB-231 at 0 or 2 min after CCL19 treatment. (**h**) Data represent mean ± s.e.m. of two independent experiments described in (**g**). Uncropped western blot images are shown in Supplementary Figs S7 and S8a. p = phosphorylated, T = total. *P < 0.05 and **P < 0.01.

Since the chemotaxis of mDCs is mainly regulated via CCR7-CCL19 interaction, we examined whether breast cancer derived soluble factors increase the expression levels of CCR7 on mDCs. Unexpectedly, the levels of CCR7 expression on mDC-MCF-7, mDC-MDA-MB-436, and mDC-SK-BR-3 were not critically affected while its level was significantly decreased on mDC-MDA-MB-231 compared with that on mDC-MCF-10A (Fig. 2e,f). To further understand the highly enhanced directionality observed in the CCL19-induced chemotaxis of mDC-MCF-7, mDC-MDA-MB-231 or mDC-MDA-MB-436, in spite of the reduced or not affected CCR7 expression, their underlying mechanisms were investigated. Based on the previous studies showing the involvement of MAP kinases, such as ERK1/2, p38 and JNK in CCR7-mediated chemotaxis of DCs, independent of Rho pathways responsible for cell velocity^29^,^30^, activation of MAP kinases and Rho was analyzed before and after CCL19 treatment. Upon CCL19 stimulation, we observed highly enhanced levels of phosphorylated (p)-JNK and p-c-Jun in mDC-MDA-MB-231 and mDC-MDA-MB-436 compared with those in mDC only or mDC-MCF-7 (Fig. 3a,b). Again, the expression level of p-JNK/total (T)-JNK or p-c-Jun/T-c-Jun was higher only in mDC-MDA-MB-231 and mDC-MDA-MB-436 (Fig. 3c,d). The expression level of p-ERK/T-ERK was comparable in all groups (Fig. 3e), and p-38/T-38 expression was slightly higher in mDC-MDA-MB-231 (Fig. 3f). In the absence of CCL19 treatment, the expression levels of p-JNK/T-JNK, p-c-Jun/T-c-Jun, p-ERK/T-ERK and p-38/T-38 were comparable among the groups (Fig. 3c–f), suggesting that the enhanced JNK/c-Jun signaling pathway observed in mDC-MDA-MB-231 or mDC-MDA-MB-436 is dependent on CCL19 stimulation. We then compared the activity of RhoA, responsible for cell velocity, between mDC only and mDC-MDA-MB-231 in the presence or absence of CCL19, and found its expression levels were comparable between the two groups (Fig. 3g,h), consistent with our similar velocity data of all groups (Fig. 2a). These results suggest that the highly increased JNK/c-Jun signaling pathway in mDC-MDA-MB-231 or mDC-MDA-MB-436 is closely correlated with their enhanced directional movement toward CCL19.

### Inhibition of JNK signaling in mDC-MDA-MB-231 abolishes their enhanced directionality toward CCL19

**Figure 4:**
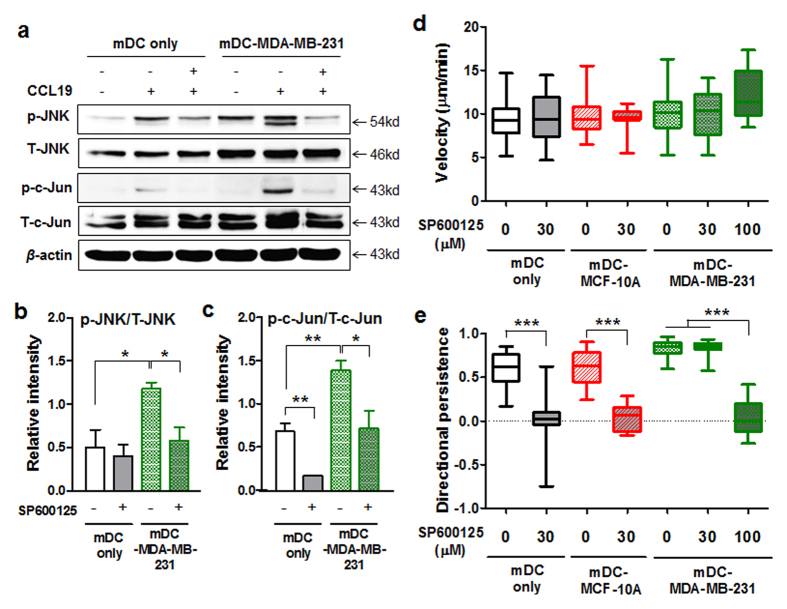
The enhanced directionality of mDC-MDA-MB-231 toward CCL19 is significantly decreased by the inhibition of JNK activity. (**a**) The expressions of JNK and c-Jun molecules in mDC only or mDC-MDA-MB-231 in the presence or absence of a 30 μM JNK inhibitor (SP600125) with or without CCL19 treatment. (**b**,**c**) Data represent mean ± s.e.m. of the ratio of p-JNK/T-JNK or p-c-Jun/T-c-Jun from three independent experiments described in (**a**). Uncropped western blot images are shown in Supplementary Fig. S8b. p = phosphorylated, T = total. (**d**,**e**) Box-and-whisker plots of the velocity (**d**) and directional persistence (**e**) of mDC only or mDC-MDA-MB-231 treated with various concentrations (0, 30 or 100 μM) of SP600125. *n* ≥ 40. *P < 0.05, **P < 0.01 and ***P < 0.001.

To further confirm the data demonstrating that the JNK signaling pathway regulates the increased directional persistence of mDC-MDA-MB-231, we applied inhibitors of ERK, JNK, or p38 to mDC only or mDC-MDA-MB-231 followed by CCL19 treatment. The appropriate activity of a JNK inhibitor was confirmed by the markedly decreased expression of p-c-Jun/T-c-Jun in both mDC only and mDC-MDA-MB-231 (Fig. 4a,c). Interestingly, the formerly enhanced expressions of p-JNK/T-JNK and p-c-Jun/T-c-Jun in mDC-MDA-MB-231 upon CCL19 stimulation was reduced to the level of those in mDC only after the JNK inhibitor treatment (Fig. 4a–c).

In our 3D microfluidic device, we found no significant difference between the migratory velocities of mDC only and mDC-MDA-MB-231 after the JNK inhibitor treatment (Fig. 4d). However, the inhibition of JNK activity was sufficient to completely abolish the increased directionality of mDC-MDA-MB-231 toward CCL19 (Fig. 4e). Interestingly, a higher concentration of the JNK inhibitor was required to suppress the enhanced directionality of mDC-MDA-MB-231 compared with that of mDC only (Fig. 4e), suggesting that the up-regulated JNK signaling (Fig. 3a,c) is crucial for the increased chemotaxis of mDC-MDA-MB-231. In 2D transwell assays with the JNK inhibitor, we again confirmed that the formerly increased migration of mDC-MDA-MB-231 toward CCL19 was dramatically decreased to the level slightly higher than that of mDC only (Supplementary Fig. S5a). In the case of p38 inhibitor assay, we observed decreased migration of mDC-MDA-MB-231 toward CCL19, but the level of reduction was much smaller than that caused by the JNK inhibitor (Supplementary Fig. S5b–d). We observed no significant effects of ERK inhibition on DC migration (Supplementary Fig. S5e–g). The inhibitors were not toxic to DCs at the concentrations we treated (Supplementary Fig. S6a,b), and there was no effect of the JNK inhibitor on the level of CCR7 expression on mDCs (Supplementary Fig. S6c,d). Taken together, these data clearly indicate that MDA-MB-231- or MDA-MB-436-derived soluble factors significantly increase the CCL19 induced chemotaxis of DCs in a JNK signaling dependent manner.

### mDC-MDA-MB-231 secrete pro-inflammatory cytokines and induce highly proliferative and activation-induced cell death (AICD)-resistant inflammatory T cells

**Figure 5:**
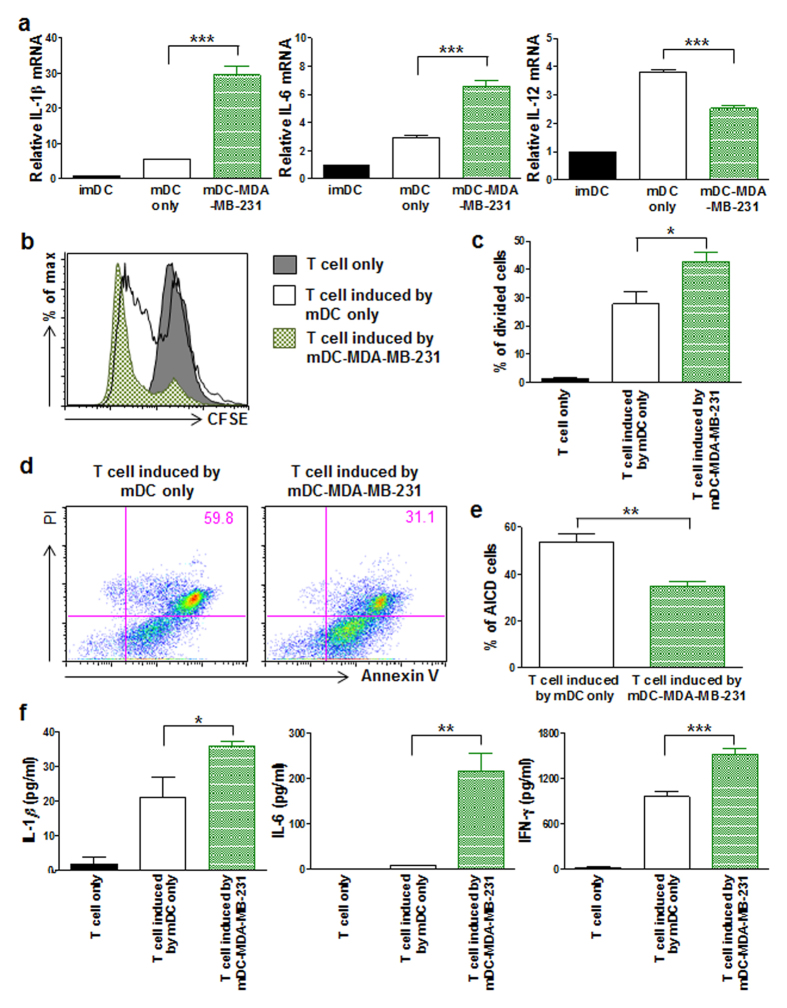
mDC-MDA-MB-231 expressing pro-inflammatory cytokines induces T cells to more proliferate and resistant to activation-induced cell death. (**a**) The relative mRNA expressions of *IL-1β*, *IL-6*, and *IL-12* in imDC, mDC only or mDC-MDA-MB-231. (**b**) Flow cytometric analysis of carboxyfluorescein succinimidyl ester (CFSE)-labeled CD3^+^ T cell only and CFSE-labeled CD3^+^ T cells co-cultured either with mDC only or mDC-MDA-MB-231. (**c**) Data represent mean ± s.e.m. of three independent experiments described in (**b**). (**d**) Flow cytometric analysis of propidium iodide (PI) and annexin V staining of CD3^+^ T cells activated by CD3 and CD28 antibodies in the presence of IL-2; these cells had previously been co-cultured either with mDC only or mDC-MDA-MB-231. (**e**) Data represent mean ± s.e.m. of three independent experiments described in (**d**). (**f**) The cytokine levels of IL-1β, IL-6 and IFN-γ secreted from CD3^+^ T cells only and CD3^+^ T cells co-cultured either with mDC only or mDC-MDA-MB-231. Data represent mean ± s.e.m. of three independent experiments (**a,f**). *P < 0.05, **P < 0.01 and ***P < 0.001.

Next, we questioned how mDC-MDA-MB-231 affects DC-mediated T cell immune responses. Cytokines secreted from DCs play a crucial role in inducing T cell immune responses, which may result in pro- or anti-tumor immune responses^31^,^32^, thus we compared cytokines from mDC only and mDC-MDA-MB-231. We observed the slightly decreased level of cytokine IL-12 and markedly increased levels of pro-inflammatory cytokines IL-1β and IL-6 in mDC-MDA-MB-231 compared with those in mDC only (Fig. 5a).

To analyze how inflammatory DCs affect CD3^+^ T cell functions, we performed an allogenic mixed lymphocyte reaction, and observed significantly enhanced proliferation of CD3^+^ T cells that had been previously co-cultured with mDC-MDA-MB-231 compared with that of CD3^+^ T cells co-cultured with mDC only (Fig. 5b,c). Moreover, these highly proliferative allogenic T cells that had been induced by mDC-MDA-MB-231 were more resistant to AICD (Fig. 5d,e), and secreted significantly higher levels of inflammatory cytokines, such as IL-1β, IL-6 and IFN-γ (Fig. 5f). Taken together, these data show that MDA-MB-231-derived soluble factors affect DCs in such a way that these DCs not only up-regulate inflammatory cytokines, but also induce inflammatory CD3^+^ T cells that are more proliferative and less susceptible to AICD, leading to chronic inflammatory milieu, which may result in promotion of tumor growth.

## Discussion

In this study, our 3D microfluidic device allows us to observe novel effects of various breast cancer cell lines on the chemotaxis of DCs toward CCL19. Interestingly, our results demonstrated a novel aspect of how the soluble factors of metastatic triple negative breast cancers (TNBCs, MDA-MB231 and MDA-MB-436) exploit DCs, leading to chronic inflammation. TNBC-derived soluble factors enhanced the CCL19 induced chemotaxis of mature DCs in a JNK signaling pathway dependent manner and generated inflammatory DCs which induce highly proliferative and less apoptotic inflammatory T cells, resulting in high inflammatory milieu, of which condition may be positively correlated with breast cancer progression^33^.

Our findings provide novel insights into the various effects of tumor microenvironments on DC function. It is well-known that appropriate regulation of maturation and migration of DCs upon foreign stimulation is the first critical step in inducing antigen-specific adaptive immune responses^34^. Moreover, the quantity and quality of antigen-specific CD4^+^ T cell responses have been shown to be proportional to the migrated number of antigen-pulsed DCs into the secondary lymphoid organs^35^, suggesting that any hindrance in DC migration may be detrimental to induce anti-tumor responses. Therefore, it is not surprising that tumors evade host immunity by impairing the immunological function of DCs^12^,^36^. It is noteworthy that our study, suggesting the enhanced CCL19-induced chemotaxis of TNBC-affected DCs, is an alternative perspective on tumor evasion strategies, where tumors may manipulate host adaptive immune responses via accelerated migration of DCs.

We identified that the CCL19 induced directional movement of TNBC derived factor-interacted DCs was increased via the enhanced JNK signaling pathway. Using our 3D microfluidic device, we clearly showed that the enhanced migratory activity of TNBC-affected DCs is due to the facilitation of directional movement rather than the velocity of DCs. This 3D multilevel microfluidic device assay provided a simple and versatile way for studying cell migration in a well-controlled *in vitro* microenvironment. The multilevel microfluidic platform does not require bulky or superfluous equipment for either fabrication or operation, allowing a low-cost, biologist-friendly, and a reliable cell-based assay (Supplementary Fig. S1)^23^. Tracking individual cells in the microfluidic device enabled us to extract meaningful features for quantitative analysis of directed movement of the cells^24^.

Interestingly, in comparison to a previous study that showed the involvement of co-operative interactions of ERK1/2, JNK and p38 in DC migration^29^, our microfluidic assay indicated that JNK inhibition alone was sufficient to completely suppress the up-regulated directional movement of mDC-MDA-MB-231 (Fig. 4e). However, since the expression level of p-p38/T-p38 was slightly increased in mDC-MDA-MB-231 (Fig. 3f) and the treatment of a p38 inhibitor resulted in the decreased chemotaxis of mDC-MDA231 toward CCL19 (Supplementary Fig. S5b–d), we do not exclude the possibility of co-operative interaction between JNK and p38 molecules in enhancing DC migration^37^. In addition, besides c-Jun, other JNK downstream molecules such as Paxillin and Spir, regulating adhesion or actin dynamics, respectively, may also have a role in cell chemotaxis of DCs^38^. In the case of breast cancer luminal types such as MCF-7 and SK-BR-3, we also observed the enhanced directionality of mDC-MCF-7 and mDC-SK-BR-3 toward CCL19. However, the enhanced chemotaxis of mDC-MCF-7 was not regulated by the JNK/c-Jun signaling pathway, thus further studies are required to investigate possible underlying mechanisms influenced by various types of breast cancer cells.

This study showed that mDC-MDA-MB-231 expressed significantly higher pro-inflammatory cytokines IL-1β and IL-6, and CD3^+^ T cells induced by mDC-MDA-MB-231 highly secreted profound pro-inflammatory cytokines, IL-1β, IL-6 and IFN-γ, and were highly proliferative and resistant to AICD. It is well-known that a chronic inflammatory niche in the host plays pivotal roles in the development and progression of various tumors^39^. Previous studies demonstrated that increased IL-6 and IL-1β cytokines are correlated with tumor invasiveness and worse prognosis, particularly in metastatic breast cancer patients^31^,^40^,^41^. Another recent study also demonstrated that IL-6 cytokine can promote the growth of triple negative breast cancer cells and support their chemotherapy resistance or resistance to apoptosis^42^. In addition, evolutionary walks driven by IFN-γ can be adapted by tumor cells to lead tumor editing and selection of resistant clones, thereby promoting the tumor growth or development^43^. In contrast to previous reports demonstrating suppressed or dysfunctional T cell activity in various tumor microenvironments^44^, our results implicated stimulatory and inflammatory T cells, indicating an alternative point of view for understanding the diversity and complexity of tumor microenvironments manipulating host anti-tumor immunity. However, we do not exclude the possibility of IFN-γ secreted from cytotoxic CD8^+^ T cells responsible for tumor rejection as well as other roles of IL-1β and IL-6 cytokines in complex immune networks^31^,^45^. Further studies are necessary to identify specific T cell subsets with distinct cytokine profiles induced by TNBC-affected DCs.

Previous studies reported that several soluble factors such as VEGF, IL-6, IL-10 and TGF-β secreted from different types of cancers may induce dysfunctional DCs^46^,^47^. In addition to those factors, we screened other possible breast cancer-derived factors that may be responsible for the enhanced directionality of mDC-MDA-MB-231 in a JNK signaling dependent manner, and found IL-1β and IL-6 cytokines, known to be related in enhancing the JNK signaling pathway^48^,^49^, are highly expressed in MDA-MB-231 and MBA-MB-436 compared with those in MCF-10A or MCF-7. However, when we suppressed the expression level of each or both cytokines in MDA-MB-231 by half via utilizing each cytokine specific shRNAs, neither the formerly enhanced chemotaxis nor the JNK expression of mDC-MDA-MB-231 was critically affected (Data not shown). Further studies are required to examine the effects of various and combined breast cancer cell-derived soluble factors on DC migration as well as following underlying mechanisms in DCs to better understand the enhanced DC chemotaxis.

In conclusion, our study proposed a new tumor evasion strategy elucidating how the soluble factors of TNBCs manipulate DCs to induce dysfunctional T cells that may support tumor development. Future preclinical studies may provide a rationale for blocking the JNK signaling pathway in DCs of metastatic breast cancer patients to reduce impaired DC-mediated immune responses, and also may suggest new insight in understanding the effects of the tumor microenvironment on immune responses.

## Methods

### Design and fabrication of microfluidic devices

A microfluidic device was fabricated for the on-chip chemotaxis assays. The microfluidic device had three different levels—a 100 μm high main channel, two 200 μm high side-channels adjacent to the main channel, and two 300 μm high side-chambers for loading media. The width of each channel was 1 mm. The multilevel microfluidic device was fabricated by using triple-layered adhesive tape as a master for molding PDMS (Supplementary Fig. S1). The tapes were cut into the desired design for each level using a cutting plotter (CE3000-60; Graphtec Corp) and stacked layer-by-layer. A 10:1 mixture of the PDMS prepolymer and the curing agent (Sylgard 184 Silicone Elastomer Kit; Dow Corning) was poured onto the master, and cured in an oven at 65 °C for 2 h. The PDMS was peeled off from the master, and diced. After holes were punched, the PDMS structure was bonded onto a 25 mm-diameter glass coverslip by oxygen plasma treatment of both the PDMS and the glass surfaces.

### Preparation of human immature dendritic cells

Peripheral blood mononuclear cells (PBMCs) were isolated from the blood of healthy donors obtained from Ulsan Blood Center, Ulsan, South Korea, by density gradient centrifugation on Ficoll-Paque Plus (GE Healthcare) according to Institutional Review Board of the Ethics Committee (IRB: 02-2011-001.) CD14^+^ monocytes were then isolated by positive selection using magnetic-activated cell sorting (MACS; Miltenyi Biotec). CD14^+^ monocytes were cultured in RPMI 1640 medium with 5% FBS, 1% penicillin/streptomycin, 1000 units/ml GM-CSF (Invitrogen), and 1000 units/ml IL-4 (Invitrogen) for 72 h, and then the same concentrations of GM-CSF and IL-4 were added for an additional 48 h.

### Cell line maintenance

Epithelial MCF10A, luminal MCF7, metastatic triple negative breast cancer MDA-MB-231 or MDA-MB-436, and HER2-overexpressing SK-BR-3 cells were purchased from the American Type Culture Collection. MCF-10A cells were cultured in DMEM/F12 media with 5% horse serum, 1% antibiotic-antimycotic solution, EGF (20 ng/ml), hydrocortisone (500 ng/ml), and insulin (10 μg/ml). MCF-7, MDA-MB-231 and MDA-MB-436 cells were cultured in RPMI 1640 medium with 5% FBS and 1% antibiotic-antimycotic solution, and SK-BR-3 cells were cultured in DMEM medium with 10% FBS and 1% antibiotic-antimycotic solution. The cells were maintained at 37 °C in a 5% CO_2_ humidified incubator. When cancer cells were co-cultured with DCs, we utilized the conditioned media, RPMI 1640 medium with 5% FBS, EGF (3.3 ng/ml), hydrocortisone (83 ng/ml), insulin (1.67 μg/ml) and 1% antibiotic-antimycotic solution.

### Co-culture of DCs with tumor cells *in vitro*

Each of various cancer cell lines was seeded in a 6-well plate at 0.5 × 10^6^ cells per well 12 h before the co-culture experiment. Then, a transwell insert with a 0.4-μm pore size membrane was placed onto the plate, and 1 × 10^6^ immature DCs were loaded into the upper well of the transwell chamber. The immature DCs were co-cultured with each of cancer cell lines for 48 h in the presence of 25 μg/ml poly (I:C) (Invivogen). Immature DCs cultured alone in the transwell chamber with 25 μg/ml poly(I:C) were used as control (mDC only).

### Microfluidic cell migration assays

The PDMS–glass devices were sterilized with 70% ethanol under a laminar flow hood with ultraviolet exposure for more than 30 min. 3 mg/ml purified bovine collagen solution (PureCol; Advanced BioMatrix) containing DCs was injected into the main channel, followed by polymerization in a 5% CO_2_, 37 °C incubator, for around 45 min. 1% agarose (Low gelling temperature agarose; Sigma–Aldrich Corp) solution was then injected into the side-channels adjacent to the main channel. After gelation of the agarose at room temperature, 3 μg/ml CCL19 (R&D Systems) in cell culture medium and blank cell culture medium were loaded into the respective side-chambers (Fig. 1a). The devices were mounted in a custom-built live cell chamber, in which 6 devices could be imaged using an EMCCD camera (Andor iXon 897; Till Photonics GmbH) under controlled temperature and CO_2_ conditions, on the motorized stage of an inverted microscope (IX81-ZDC; Olympus Corp). The devices were incubated for 2 h in the live cell chamber to allow formation of a linear CCL19 gradient across the collagen gel and to allow the cells to stabilize. For MAP kinase inhibition experiments on-chip, we utilized DCs (0.25 × 10^6^ cells) that had been previously co-cultured with or without MDA-MB-231 (0.5 × 10^6^ cells) in the presence of poly (I:C) for 2days in our co-culture system, and inhibitors of JNK (SP600125, 30 or 100 μM), p38 (PD98059, 20 μM), or ERK (U0126, 10 μM), purchased from Calbiochem, were added to the collagen gel prior to mixing with the DCs. To maintain the concentrations of each inhibitor in the microfluidic channel, media mixed with particular concentrations of each inhibitor were loaded into the side-chambers. Time-lapse images of the cells were taken in bright field every 2 min using a 10× objective, for a total duration of 2 h.

### Transwell cell migration assays

mDCs (2 × 10^5^ cells) were loaded into the upper well of an transwell insert with an 8-μm pore membrane (BD Biosciences) in 100 μl of media, and 600 μl of media with 100 ng/ml CCL19 was added into the bottom well of the insert. After 4 h of incubation in a 37 °C incubator, the number of mDCs that had migrated to the bottom well was counted by flow cytometry. For the inhibitor assays, mDC only or mDC-MDA-MB-231 was treated with the inhibitor of JNK (SP600125, 30 μM), p38 (PD98059, 20 μM) or ERK (U0126, 10 μM) for 1 h in a 37 °C incubator, and the transwell migration assay was performed as described before with the inhibitor present in both the upper and bottom wells of the insert.

### Flow cytometry

Cells were washed with fluorescent-activated cell sorting (FACS) buffer (2% FBS in PBS) twice, and were then stained with antibodies at 4 °C for 30 min. Data were acquired using a FACS Calibur or LSRfortessa (BD Biosciences) and analyzed with a FlowJo software (Tree Star Inc., Ashland, OR). Median fluorescence intensity (MFI) data were shown after the subtraction of corresponding isotype control values. Antibodies used in flow cytometry as follows: CD11c (B-ly6), CD80 (L307.4), CD83 (HB15e), CD86 (IT2.2), and HLA-DR (TU36) were purchased from BD Biosciences, CCR7 (3D12) and Rat IgG2a were purchased from eBioscience, and CD3 (HIT3a) and CD28 (CD28.2) were purchased from BioLegend.

### Western blotting

mDC only, mDC-MCF-7, mDC-MDA-MB-231 and mDC-MDA-MB-436 were treated with 100 ng/ml CCL19 for 0 or 30 min in a 37 °C incubator, followed by western blotting (WB) assays. For inhibitor tests, mDC only or mDC-MDA-MB-231 was treated with each of 30 μM JNK, 10 μM ERK or 20 μM p38 inhibitor for 1 h, and then the cells were treated with 100 ng/ml CCL19 for an additional 30 min in a 37 °C incubator. Antibodies used in western blotting as follows: p = phosphorylated, T = total, p-ERK1/2 (Thr202/Tyr204), T-ERK1/2 (137F5), p-JNK (Thr183/Tyr185) and p-p-38 (Thr180/Tyr182) were purchased from Cell Signaling, T-JNK (37/pan-JNK/SAPK1) and T-p38 (27/p38α/SAPK2a) were purchased from BD biosciences, p-Jun (KM-1) and T-Jun (H-79) were purchased from Santa Cruz, and β-actin was purchased from Ambion. GTP-RhoA was blotted using a Rho Activation Assay Biochem kit (Cytoskeleton). Briefly, mDC only or mDC-MDA-MB-231 was treated with 100 ng/ml CCL19 for 0 or 2 min. The cells were immediately lysed and then incubated with rhotekin-RBD beads to pull down GTP-RhoA molecules, followed by a western blot assay.

### Allogenic mixed lymphocyte reaction

CD3^+^ T cells were isolated from peripheral blood mononuclear cells by using a pan T cell isolation cocktail kit (Miltenyi Biotec) and MACS. CD3^+^ T cells (1 × 10^5^ cells) labeled with carboxyfluorescein succinimidyl ester (CFSE, Invitrogen) were co-cultured either with 0.2 × 10^5^ cells of mDC only or mDC-MDA-MB-231 for 4 days, and the percentage of divided cells was measured by flow cytometry. For the activation-induced cell death (AICD) assay, CD3^+^ T cells that had been previously co-cultured either with mDC only or mDC-MDA231 were purified and allowed to rest for 1 day in the presence of 50 units/ml IL-2 (BioLegend). Then, the CD3^+^ T cells were stimulated with plate-bound anti-CD3 (5 μg/ml, BioLegend) and soluble anti-CD28 (1 μg/ml, BioLegend) antibodies in the presence of 50 units/ml IL-2 for additional 2 days. The stimulated CD3^+^ T cells were stained with PI (BD Bioscience) and annexin V (BD Bioscience), and analyzed by flow cytometry.

### Quantitative RT-PCR analysis

CD3^+^ T cells were co-cultured either with mDC only or mDC-MDA-MB-231 and then isolated by magnetic-activated cell sorting (MACS). Total RNA was extracted with Trizol (Invitrogen), and cDNA was synthesized using the cDNA Synthesis kit (NEB). A Light Cycler 480 II (Roche) was used to perform a SYBR Green-based qRT-PCR experiment. The house-keeping gene, *GAPDH,* was used for normalization. The qRT-PCR primers are as follows: human IL-1β, forward 5′-ACATCAGCACCTCTCAAGCA-3′ reverse 5′-AGTCCACATTCAGCACAGGA-3′; human IL-6, forward 5′-CTGGTCTTTTGGAGTTTGAGGT-3′ reverse 5′-GGAACTGGATCAGGACTTTTGT-3′; human IL-12, forward 5′-CCACAAAAATCCTCCCTTGA-3′ reverse 5′-AGGGACCTCGCTTTTTAGGA-3′; human GAPDH, forward 5′-TGACAACTTTGGTATCGTGGA-3′ reverse 5′-CAGTAGAGGCAGGGATGATGT-3′. Melting temperature was 60 °C.

### Quantification of cytokines

Various cytokines in cultured supernatants were measured using a Spectramax Plus 384 (Molecular Devices) following a sandwich-based ELISA protocol (BioLegend).

### Statistical analysis

Results are expressed as mean ± s.e.m. Data normality was tested with Lilliefors test. If the data have a normal distribution, Student’s *t*-test was used to identify statistically significant differences between the groups. Otherwise, the Mann-Whitney test was used. *P*-values < 0.05 were considered as statistically significant: ^*^P < 0.05, ^**^P < 0.01, and ^***^P < 0.001. Microsoft Excel and Prism Graphpad were used for the statistical analysis and graphing.

## Additional Information

**How to cite this article**: Hwang, H. *et al.* Human breast cancer-derived soluble factors facilitate CCL19-induced chemotaxis of human dendritic cells. *Sci. Rep.* **6**, 30207; doi: 10.1038/srep30207 (2016).

## Supplementary Material

Supplementary Information
