# Supplementary material for: Human breast cancer-derived soluble factors facilitate CCL19-induced chemotaxis of human dendritic cells
Source: Sci Rep. 2016 Jul 25;6:30207. doi: 10.1038/srep30207 (PMC4958978; doi:10.1038/srep30207)
Supplement: Supplementary Information [file srep30207-s1.pdf]

Supplementary Information

Human breast cancer-derived soluble factors facilitate CCL19-induced chemotaxis of human dendritic cells

Hyundoo Hwang,[1†] Changsik Shin,[2†] Juhee Park,[2†] Enoch Kang,[1,3] Bongseo Choi,[4] Jae-A Han,[4] Yoonkyung Do,[4] Seongho Ryu,[5*] Yoon-Kyoung Cho[2,4,*]

[1]*School of Engineering and Sciences, Tecnológico de Monterrey, Av. Eugenio Garza Sada 2501 Sur, Monterrey, NL 64849, Mexico;*

[2]*Center for Soft and Living Matter, Institute for Basic Science (IBS), UNIST-gil 50, Ulsan 44919, Republic of Korea;*

[3]*Department of Bio and Brain Engineering, KAIST, 291 Daehak-ro, Yuseong-gu, Daejeon 34141, Republic of Korea;*

[4]*School of Life Sciences, Ulsan National Institute of Science and Technology (UNIST), UNIST-gil 50, Ulsan 44919, Republic of Korea;*

[5]*Soonchunhyang Institute of Medi-bio Science (SIMS), Soonchunhyang University, Chonan-Si Chungcheongnam-do 31538, Republic of Korea*

†H.H., C.S., and J.P. contributed equally to this work.

***To whom correspondence should be addressed:** Yoon-Kyoung Cho[2,4] or Seongho Ryu[5]

Email: ykcho@unist.ac.kr or ryu@sch.ac.kr

Keywords: breast cancer | chemotaxis | dendritic cells | microfluidics | inflammation

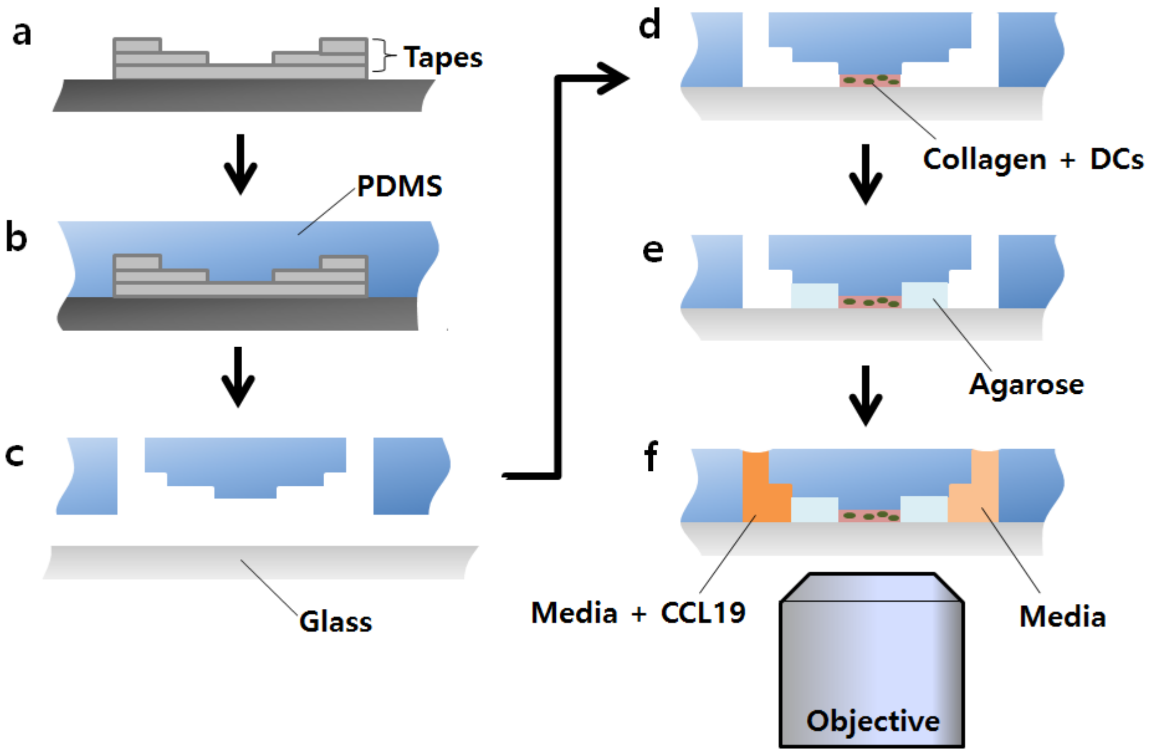

Figure S1. Fabrication process for preparing a multilevel microfluidic device for cell migration studies. (**a**) Adhesive tape was cut into a desired design for each level using a cutting plotter and the sections stacked to construct multilevel structures. (**b**) A 10:1 mixture of PDMS prepolymer and curing agent was poured onto the tape master, followed by curing in an oven at 65℃ for 2 h. (**c**) The PDMS was peeled off from the master, diced into individual chips, and holes were punched. The PDMS mold was bonded onto a glass coverslip after oxygen plasma treatment of both surfaces, followed by sterilization with 70% ethanol and UV exposure for 30 min. (**d**) A collagen gel containing DCs was injected into the shallowest main channel, followed by polymerization in a 5% CO_2, 37℃ incubator for 50 min. (**e**) Then, 1% agarose solution was injected into the channels adjacent to the main channel, followed by gelation of the agarose solution at room temperature. (**f**) Culture medium with CCL19 (3 μg/ml) or culture medium only was loaded into the individual chamber, to generate the linear concentration gradient of CCL19 across the collagen gel.

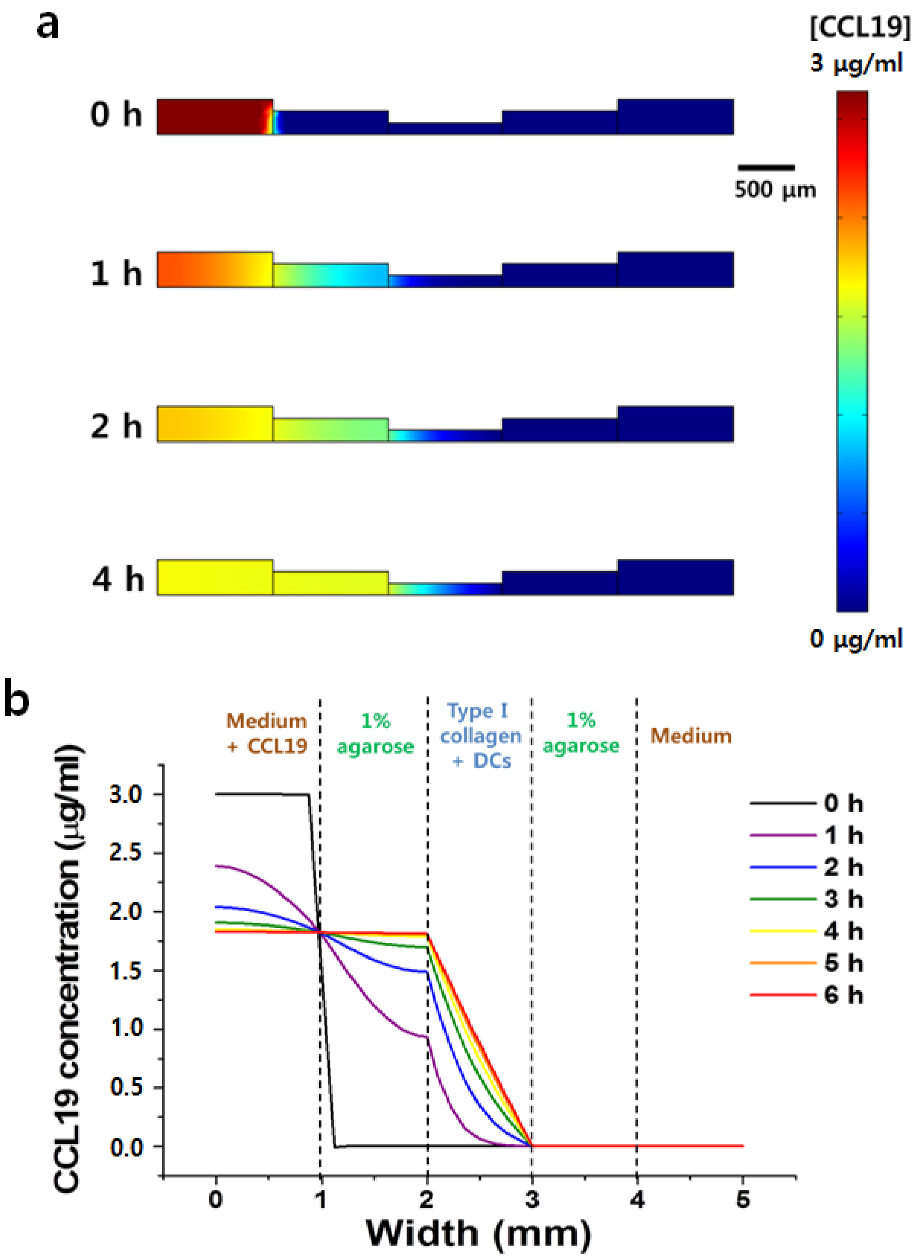

Figure S2. Generation of a linear chemical concentration gradient. (**a**) Simulation results showing the CCL19 concentration distribution in the microfluidic device over time. (**b**) Plots showing the temporal change in the CCL19 concentration profile across the microchannels.

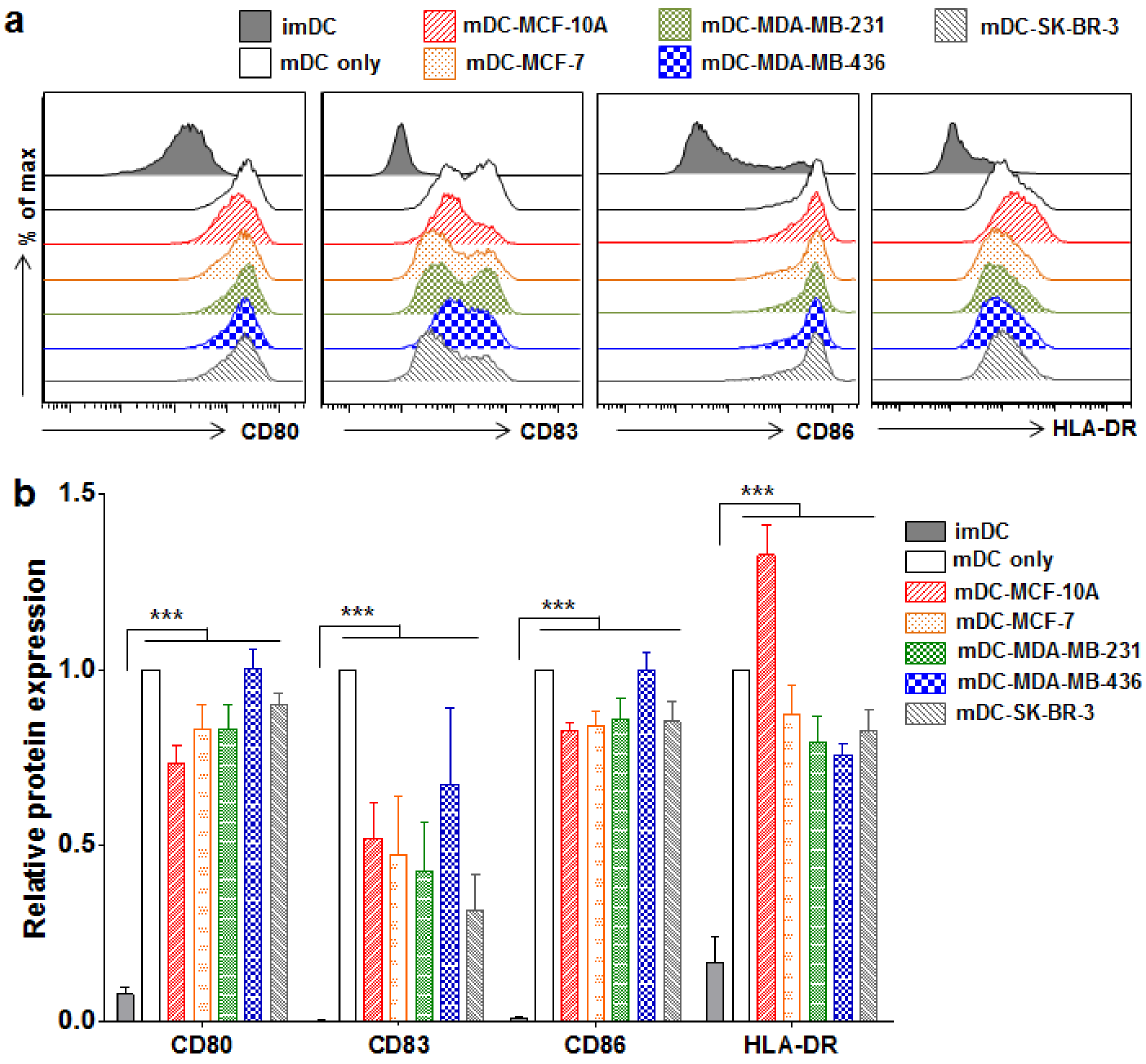

Figure S3. Expression of maturation markers on DCs. (**a**) Flow cytometric analysis of maturation markers on immature DCs (imDC), mature DCs (mDC only), or mDCs co-incubated with MCF10A (mDC-MCF-10A), MCF7 (mDC-MCF-7), MDA-MB-231 (mDC-MDA-MB-231), MDA-MB-436 (mDC-MDA-MB-436) or SK-BR-3 (mDC-SK-BR-3) cells in the presence of poly (I:C). (**b**) Data represent mean ± s.e.m. of more than five independent experiments. Data was normalized by mDC only (value=1). *** $P < 0.001$.

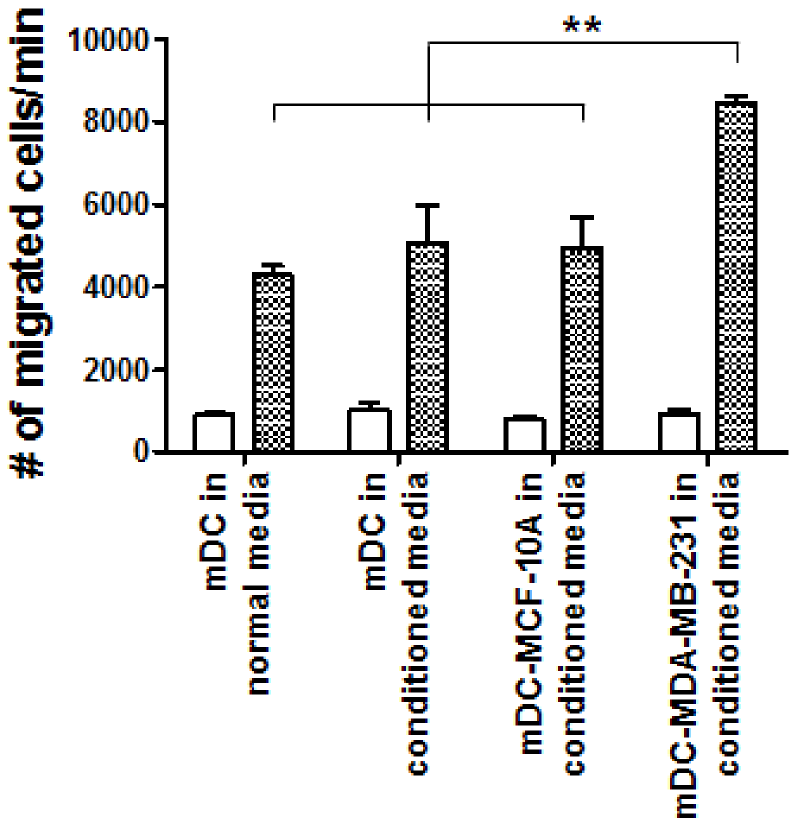

Figure S4. A transwell migration assay for CCL19-induced chemotaxis of mDCs. The number (#) of migrated mDCs cultured under the indicated conditions toward CCL19. Data represent mean ± s.e.m. of three independent experiments. Normal media is RPMI 1640 medium with 5% FBS and 1% antibiotic-antimycotic solution and conditioned media is RPMI 1640 medium with 5% FBS, EGF (3.3 ng/ml), hydrocortisone (83 ng/ml), insulin (1.67 µg/ml) and 1% antibiotic-antimycotic solution. ***P* < 0.01.

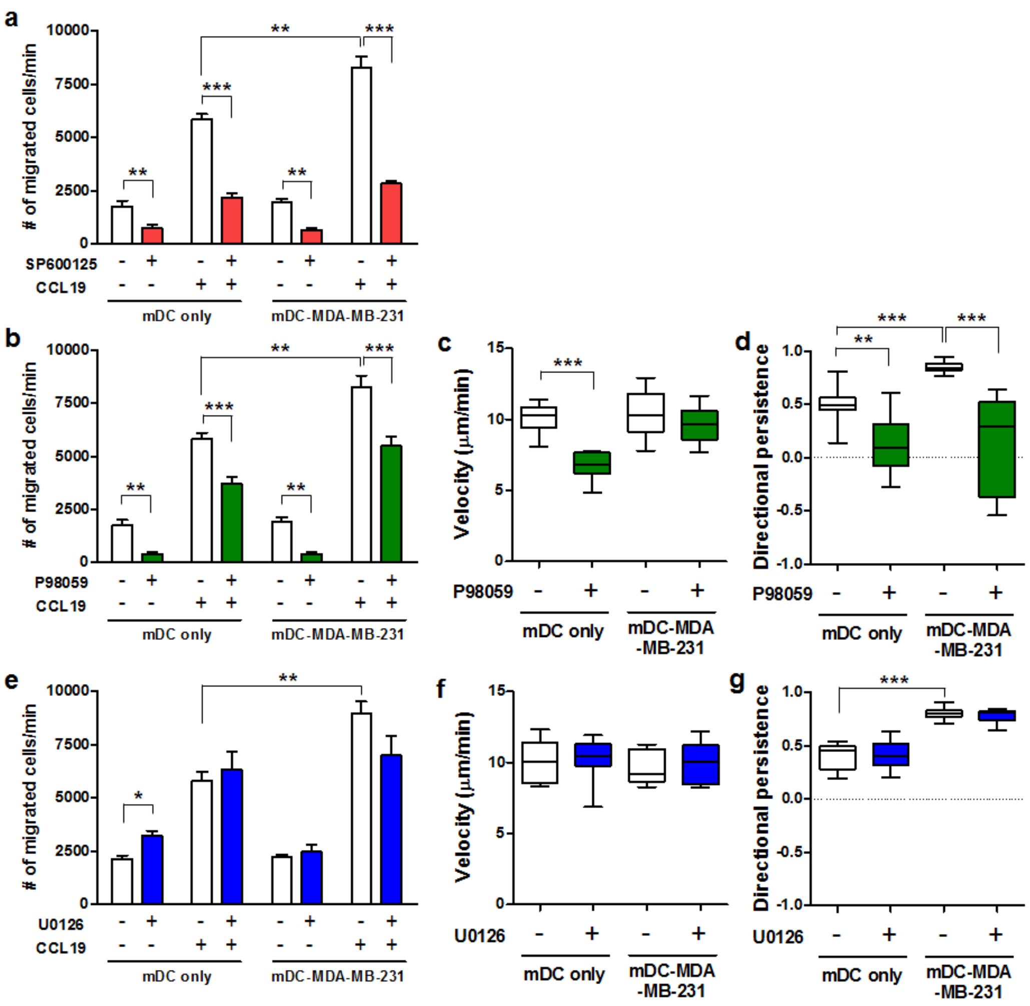

Figure S5. Effect of inhibitors on CCL19-induced chemotactic activity of mDCs in transwell or microfluidic chemotaxis assays. The migrated number (#) of mDC only or mDC-MDA-MB-231 in the presence or absence of CCL19 after treatment with a JNK (**a**), p38 (**b**) or ERK (**e**) inhibitor. Data represent mean ± s.e.m. of three independent experiments. CCL19 induced chemotaxis of mDC only or mDC-MDA-MB-231 in the 3D microfluidic channel in the presence or absence of a p38 (**c-d**) or ERK inhibitor (**f-g**). Box-and-whisker plots of the velocity and directional persistence of mDC only and mDC-MDA-MB-231 were analyzed. $n \geq 10$. $^{**}p < 0.01$ and $^{***}P < 0.001$.

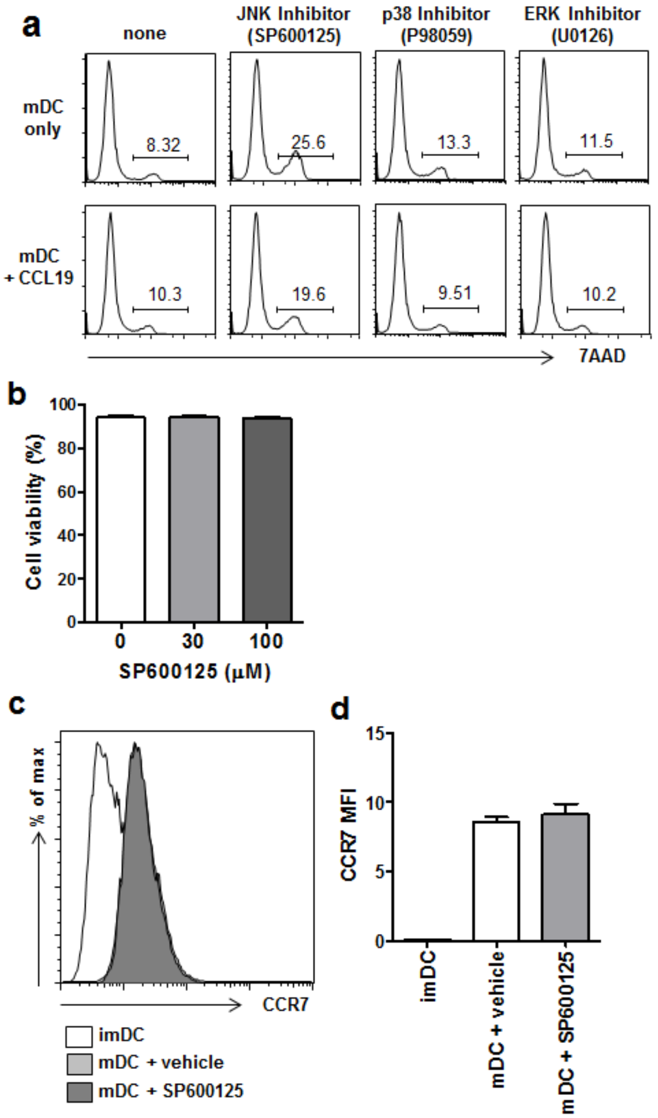

Figure S6. Inhibitor toxicity assays. (**a**) Flow cytometric analysis of inhibitor toxicity (30 µM JNK, 20 µM p38, or 10 µM ERK inhibitor) in mDC only in the presence or absence of CCL19. Data are representative of two independent experiments. (**b**) mDC only was treated with various concentrations of a JNK inhibitor (30, 50 or 100 µM SP600125), and cell viability was analyzed via a live-dead cell assay (n=3). (**c**) mDC only was treated with vehicle (DMSO) or a JNK inhibitor (30 µM SP600125) for 1 h and the expression of CCR7 on the mDCs was analyzed by flow cytometry. (**d**) Data represent mean ± s.e.m. of two independent experiments.

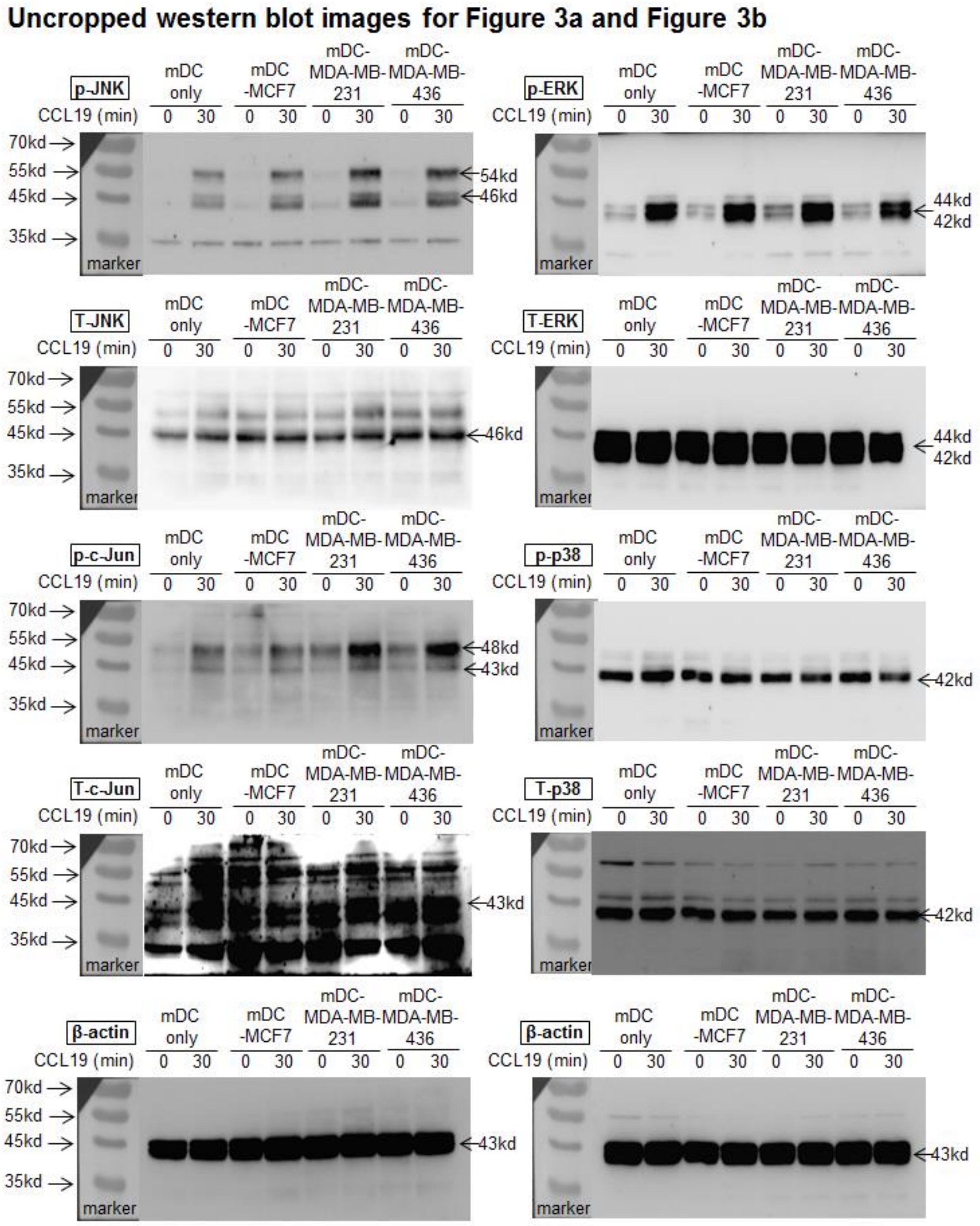

Figure S7. Full-length Western blot images for Fig. 3a,b.

Representative uncropped western blot images of MAP kinase molecules, JNK, c-Jun, ERK or p-38, in mDC only, mDC-MCF-7, mDC-MDA-MB-231 or mDC-MDA-MB-436 described in Fig. 3a,b. p=phosphorylated, T=total.

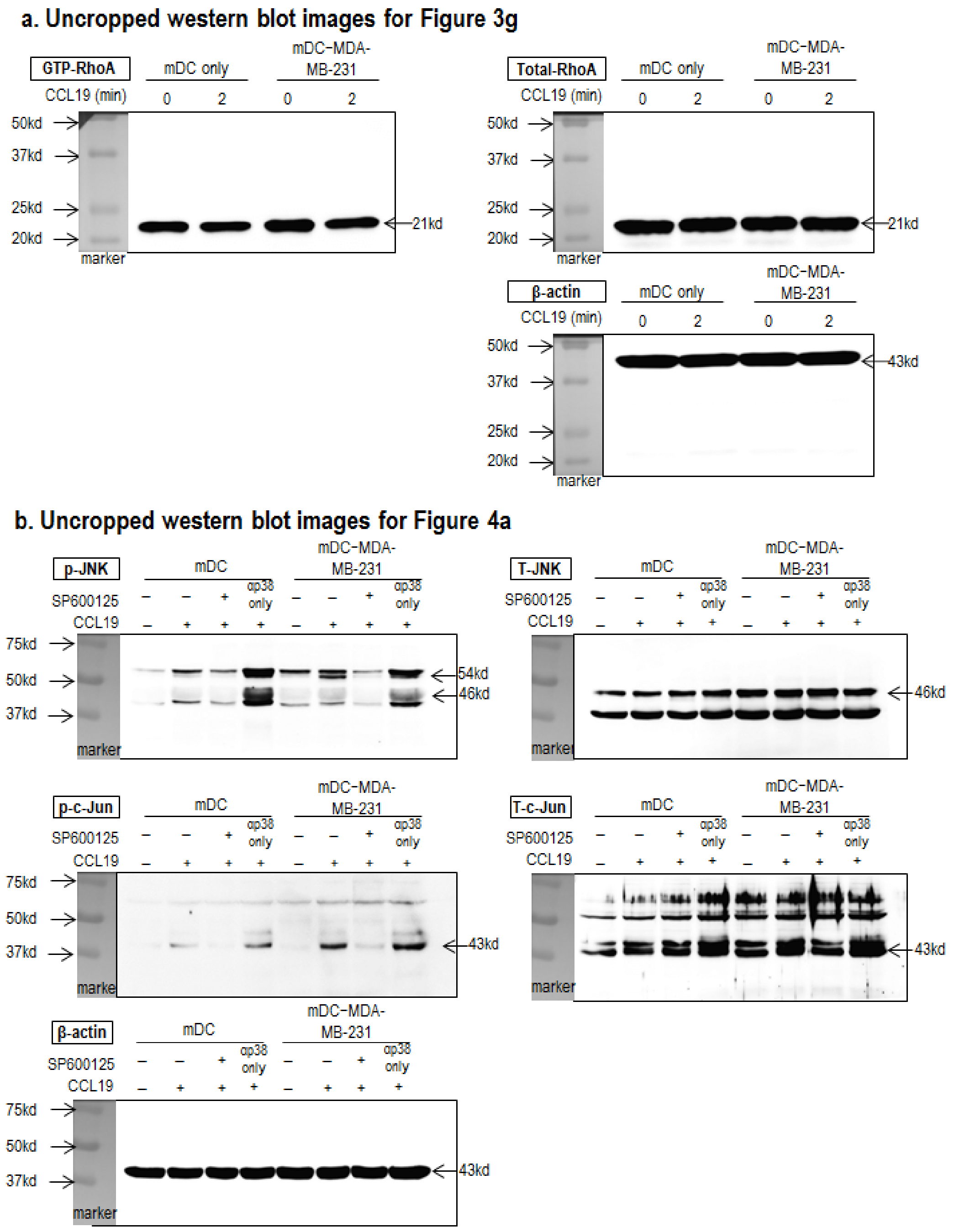

Figure S8. Full-length Western blot images for Fig. 3g and Fig. 4a

(**a**-**b**) Representative uncropped western blot images of GTP-RhoA in mDC only or mDC-MDA-MB-231 described in Fig. 3g (**a**) or of JNK and c-Jun molecules in mDC only or mDC-MDA-MB-231 in the presence of a JNK inhibitor described in Fig. 4a (**b**). p=phosphorylated, T=total.
